# Unraveling the mechanisms of nicotine-induced osteoporosis via network toxicology, bioinformatics, and molecular docking

**DOI:** 10.18332/tid/215177

**Published:** 2026-01-20

**Authors:** Song Xu, Guozhu Wang, Jiaxin Liu, Xiongwen Zhang, Xie Dong, Jin Liang, Tao Bai

**Affiliations:** 1 Orthopedic Sugery Division One (Spine&Trauma), Sixth Affiliated Hospital of Kunming Medical University, Yuxi City, China; 2 School of Medicine, Kunming University of Science and Technology, Kunming, China

**Keywords:** nicotine, osteoporosis, network toxicology, bioinformatics, molecular docking

## Abstract

**INTRODUCTION:**

Osteoporosis (OP) is linked to smoking. Nicotine may disrupt bone homeostasis through various pathways, but its molecular mechanisms are unclear. This study aims to explore the molecular networks and key regulatory factors underlying nicotine-induced OP.

**METHODS:**

Nicotine toxicity was assessed via ProTox-3.0, with its Simplified Molecular Input Line Entry System (SMILES) structure retrieved from PubChem. Potential targets were predicted using five databases, including SuperPred. OP-related gene data (GSE56815) were extracted from Gene Expression Omnibus (GEO) and combined with GeneCards and Comparative Toxicogenomics Database (CTD) for target screening. Overlapping genes were identified by Venn diagram analysis, followed by protein-protein interaction (PPI) network construction. Gene Ontology (GO) and Kyoto Encyclopedia of Genes and Genomes (KEGG) enrichment analyses were performed using HipLot, while Hallmark Gene Sets provided insights into key biological pathways. Core targets were screened via Cytoscape 3.9.1, and molecular docking was conducted using AutoDockTools 1.5.7.

**RESULTS:**

In all, 388 nicotine-associated targets and 1777 OP genes were predicted, with 116 overlapping. Enrichment analyses revealed associations with multiple signaling pathways, particularly those involving apoptosis and estrogen. Eight core targets, including SRC, BCL2, and CASP3, were identified. Molecular docking showed strong binding affinity (approximately -5 kcal/mol), with enhanced binding stability through hydrophobic interactions and hydrogen bonding.

**CONCLUSIONS:**

This study suggests nicotine exacerbates OP by regulating key targets, such as CASP3 and ESR1, and pathways like apoptosis and estrogen signaling. These findings provide insights into the molecular mechanisms underlying nicotine’s role in OP and potential therapeutic targets.

## INTRODUCTION

Nicotine, one of the most important biologically active components of tobacco, has been linked to various health problems, including nicotine addiction^1^, lung cancer^2^, neurological disorders^3^, among others. In addition to its effects on lungs, neurological function, and cancers, nicotine is also potentially hazardous to bone health. Recent studies have shown that the effects of nicotine on bone health may be realized through a variety of mechanisms, including interference with bone metabolism, inhibition of osteoblast proliferation and function, and enhancement of osteoclast activity, which promotes the onset and development of osteoporosis^4,5^.

Osteoporosis (OP) is a metabolic disease characterized by decreased bone mineral density, degradation of bone microarchitecture, and increased bone fragility, which significantly increases the risk of fracture and poses a major public health problem, especially in the elderly population^6^. The development of osteoporosis is usually closely related to a variety of factors, including age, gender, genetic background, hormone levels, and lifestyle. Among them, there is a significant and complex relationship between smoking and osteoporosis, which can cause effects from various aspects such as bone cell activity^7^, hormone metabolism^8^, and immune system^9^. Studies have shown that nicotine impairs bone health and increases the risk of osteoporosis, mainly by inhibiting osteoblast activity, promoting bone resorption^10^, inducing oxidative stress and apoptosis, and decreasing bone mineral density and bone strength^11^. However, although many studies have examined the effects of smoking and nicotine on osteoporosis, the specific role of nicotine in the pathogenesis of this condition is still not fully understood.

In recent years, the development of cybertoxicology and transcriptomics technologies has provided us with a new research platform, enabling the exploration of the effects of chemical substances on health to be analyzed from a multilevel and multidimensional perspective. Network toxicology is an emerging discipline that utilizes bioinformatics to systematically elucidate the toxicological effects of chemical substances and their roles in disease development by integrating their interactions with biological macromolecules (such as genes and proteins)^12,13^. Through network analysis, we can gain a deeper understanding of the roles and potency of molecules in the network of disease mechanisms, and ultimately can find relevant targets to prevent or treat diseases. Transcriptomics studies, on the other hand, help to reveal disease-associated genes and the roles they play in specific biological processes by analyzing gene expression data on a large scale^14^. For example, by integrating multi-organizational transcriptomic data, researchers are able to identify lncRNA regulatory networks associated with complex diseases^15^, or resolve the effects of genetic variation on gene expression through cross-population analysis^16^. By integrating these two approaches, a systematic biological basis can be provided to explore the relationship between nicotine and osteoporosis.

The aim of this study is to investigate the pathogenic mechanisms related to the effects of nicotine on osteoporosis by integrating multiple advanced techniques, including network pharmacology, transcriptomics, bioinformatics, and molecular docking. Through the combined application of these methods, this study provides theoretical support for further understanding of the mechanism of action of nicotine, and hopefully provides more precise biomarkers and therapeutic regimens for the early diagnosis and treatment of osteoporosis, as well as a solid foundation for subsequent experimental studies and clinical validation.

## METHODS

**Figure 1:**
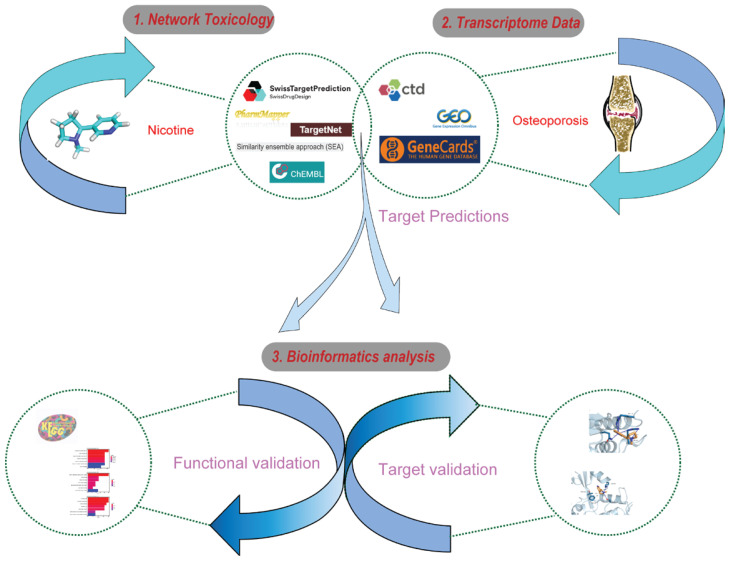
Network toxicology approach to nicotine and osteoporosis. Comprehensive flowchart methodology for studying the relationship between nicotine and osteoporosis using a network toxicology approach

A network toxicology workflow for nicotine and osteoporosis is shown in Figure 1.

### Nicotine target prediction

The databases and tools used in this study were referenced from previous literature^17,18^. First, the simplified molecular input line entry system (SMILES) structural formula of nicotine was retrieved by PubChem and analyzed using SuperPred, Target Net, PharmMapper, ChEMBL, SwissTargetPrediction and Similarity Ensemble Approach databases for potential targets of nicotine action prediction. To ensure reliability, overlapping targets obtained across different databases were identified and duplicates removed, and target information was standardized using official gene symbols. By screening the targets with high confidence, we finally obtained a set of targets that may be related to nicotine. At the same time, we tested them for toxicity using the ProTox-3.0 online database.

### Osteoporosis-related target screening

The osteoporosis-related gene expression dataset GSE56815 was obtained from the Gene Expression Omnibus (GEO). Differential expression analysis was performed using the *limma* R package (version 3.6.3) after quantile normalization of expression values to ensure comparability across samples. *Limma* fits linear models to each gene, assuming approximate normality of residuals after log2 transformation, independence of samples, and comparable variance across genes, with variance estimates stabilized through an empirical Bayes method to enhance robustness under small sample sizes. Differential expression between the osteoporosis and normal groups was evaluated using the *limma* package, with p-values adjusted for multiple testing by the Benjamini-Hochberg method to control the false discovery rate (FDR <0.05). DEGs were defined as those with log2 fold change (log2 FC) >0.5 and an adjusted p<0.05. Volcano plots were then generated to visualize the differential expression, with genes exhibiting adjusted p<0.05 considered significant. P-values for differential expression were derived from the empirical Bayes-moderated statistics implemented in the *limma* package. In addition, osteoporosis-related targets were retrieved from the GeneCards database using the keyword ‘osteoporosis’, and those with relevance scores >1.5 were selected. The Comparative Toxicogenomics Database (CTD) was also searched with the same keyword, and targets with scores >50 were included. Finally, the targets obtained from GEO, GeneCards, and CTD were integrated to generate the final set of osteoporosisrelated targets.

### PPI network construction

First, the nicotine targets were collapsed with the osteoporosis targets and their intersection, i.e. the potential targets of nicotine’s action on osteoporosis, was visualized using a Venn diagram. Then, the intersecting targets were imported into the STRING database to construct a PPI network. In the STRING database, ‘Multiple proteins’ was selected to input target genes, the species was set as ‘Homo sapiens’, and the minimum interaction threshold was set as ‘medium confidence’. The minimum interaction threshold was set to ‘medium confidence’ (0.400) in the STRING database. To improve the clarity of the network, free nodes were hidden, and only protein interactions were displayed. Finally, the results were saved as JPG and TSV files.

### GO/KEGG enrichment analysis

In order to comprehensively analyze the basic functions and signaling pathways involved in the core target genes, this study used Hiplot^19^ for Gene Ontology (GO) and Kyoto Encyclopedia of Genes and Genomes (KEGG) enrichment analysis. A statistical significance threshold was set at an adjusted p<0.05. First, the significance of enrichment in biological processes (BP), molecular functions (MF), and cellular components (CC) was determined. GO analysis helped to reveal the possible roles of core target genes by assessing their molecular functions, cellular components, and involvement in biological processes. In contrast, KEGG enrichment analysis focuses on the biological pathways involving the target genes, further elucidating their potential mechanisms in disease occurrence and development.

In order to explore the differences in gene expression under different conditions, we employed Hallmark Gene Sets from the SangerBox^20^ online platform for enrichment analysis using the R package *clusterProfiler* (version 3.14.3). The minimum gene size was set to 5 and the maximum to 5000. Values of p<0.05 and an FDR <0.2 were considered statistically significant. The intersecting genes were input, and FDR correction was applied for multiple testing correction in the Hallmark gene set enrichment analysis. Circle plots were generated to facilitate intuitive visualization. In order to gain a deeper understanding of the similarity and clustering relationships among different functional terms, this study utilized the Metascape^21^ platform to perform functional term clustering on the significantly enriched entries obtained from the enrichment analysis. Kappa similarity-based clustering was applied with a threshold of >0.3 to effectively categorize the terms. One representative term was selected from each cluster for presentation. All terms were converted into a network layout, with terms represented as circular nodes, and different clusters differentiated by color. Terms with a similarity >0.3 were connected by edges, with edge thickness reflecting the similarity strength. Finally, the network was visualized in Cytoscape using a force-directed layout, and the edge bundling technique was applied to reduce overlap and enhance readability.

### Core target analysis

The PPI network was imported into Cytoscape (version 3.9.1) for visualization and topological analysis. To identify core targets in the network, six algorithms in Cytoscape were applied: Maximal Clique Centrality (MCC), Edge Percolation Component (EPC), Maximum Neighborhood Component (MNC), Closeness Centrality, Radiality Centrality, and Degree. Ultimately, we screened the core targets in the PPI network by taking the intersection of the top ten ranked genes analyzed by these six algorithms.

### Molecular docking

To verify the binding ability of nicotine to the core target, molecular docking experiments were performed. First, the SDF file of nicotine was converted into PDB format using OpenBabel software. The crystal structures of core target proteins were retrieved from the RCSB PDB database and screened according to the following principles: the organism source was Homo sapiens, determined by X-ray diffraction as the experimental method, and with a refinement resolution of <3.0 Å. Subsequently, proteins were preprocessed by removing water molecules and any bound ligands, and adding hydrogen atoms to optimize protonation states. Then, molecular docking was conducted using AutoDock Tools 1.5.7^22^ with 20 runs to calculate the binding energy. A more stable small molecule-protein interaction results in a lower binding energy, indicating stronger affinity. When the binding free energy is ≤ -5 kcal/mol, it is considered to indicate good binding affinity^23^. Finally, the conformation with the lowest binding energy was selected, and 3D binding interaction maps were generated using PyMOL 2.4.0 software to illustrate the binding interactions of nicotine with target proteins.

### Statistical analysis

Data are presented as mean ± standard deviation (SD). For gene expression analysis, differential expression was calculated using the *limma* package, and p-values were adjusted using the Benjamini-Hochberg method for FDR correction (FDR <0.05). Statistical significance was annotated as follows: ns = not significant (p≥0.05), p<0.05 = significant, p<0.01 = very significant, and p<0.001 = extremely significant. All p-values were two-sided, and each experiment was repeated at least three times to ensure reproducibility. Data visualization was performed using R software (version 3.6.3) and Cytoscape (version 3.9.1). Volcano plots were generated with the *ggplot2* package, Venn diagrams with VennDiagram, and PPI networks with Cytoscape. Molecular docking visualization was conducted using PyMOL (version 2.4.0). The databases and tools applied in this study with their Uniform Resource Locators (URLs) are provided in Supplementary file Table 1.

## RESULTS

### Prediction of nicotine-related targets

Toxicity testing with ProTox-3.0 indicated grade 1 toxicity, suggesting potential effects on humans. Meanwhile, in order to identify the potential targets of nicotine, several target prediction tools were used in this study for analysis. The SMILE and structural map of nicotine were firstly obtained in PubChem (Supplementary file Figure 1A). Subsequently, through SwissTargetPrediction, a total of 28 potential targets were predicted; through the Target Net tool, 49 potential targets; through the ChEMBL tool, 247 potential targets; through the SuperPred database, 115 potential targets; through the PharmMapper database, 8 potential targets, and the Similarity Ensemble Approach (SEA) tool, 7 potential targets. After combining the predictions from these six databases and removing duplicates, we obtained 388 potential targets for nicotine (Supplementary file Figure 1B).

### Acquisition of relevant targets for osteoporosis

To identify targets associated with osteoporosis, we first obtained the osteoporosis dataset GSE56815 from the GEO database and systematically analyzed it. Box plot results showed that the median lines of the individual samples were located at the same level (Supplementary file Figure 2A), indicating the overall consistency of the data. Specific analyses showed a total of 3091 differentially expressed genes between the control and osteoporosis groups in the dataset (Supplementary file Figure 2B). Next, we performed a volcano plot display of the differentially expressed genes (Supplementary file Figure 1C) to visualize the differences in gene expression more closely. Then, we screened 118 genes that were differentially expressed in osteoporosis-associated samples with absolute log2 FC values >0.5. To further identify targets associated with osteoporosis, we applied GeneCards, a webbased pharmacological analysis tool, to screen 1463 osteoporosis-related genes with a score >1.5, and also screened 1069 osteoporosis-related genes with a score >50 in the CTD database. Finally, these gene results were subjected to concatenation processing and removal of duplicate values, resulting in 1777 osteoporosis-related genes (Supplementary file Figure 1D).

### Construction of protein-protein interaction network (PPI)

In order to deeply explore the mechanism of action of nicotine in osteoporosis, we first identified the intersecting targets of nicotine and osteoporosis, and obtained a total of 116 intersecting genes (Supplementary file Figure 1E). Subsequently, we imported these 116 co-interacting targets into the STRING database, constructed a PPI network, and displayed the relevant network information (Supplementary file Figure 1F).

### Enrichment analysis

To further investigate the mechanism of action of nicotine-induced osteoporosis, we performed GO and KEGG enrichment analysis of 116 potential targets via the HipLot online functional enrichment website. The results of the GO enrichment analysis showed that the 116 targets involved 3805 biological process (BP), 269 cellular composition (CC), and 471 molecular function (MF) entries. We plotted GO (BP), GO (CC), and GO (MF) function analysis bubble maps based on the top 7 entries in terms of count number (Supplementary file Figure 3A).

The KEGG pathway enrichment analysis results showed that these 116 targets were associated with 253 signaling pathways. We selected the top 7 signaling pathways in terms of count number and plotted the bar graphs (Supplementary file Figure 3B), and these KEGG pathways were involved in the following main areas: the effect of nicotine on osteoporosis, lipid metabolism and atherosclerosis, apoptosis, hepatitis B virus infection, prostate cancer, fluid shear stress and atherosclerosis, and human cytomegalovirus infection. Specifically, nicotine may be closely related to the development and progression of diseases such as atherosclerosis, cancer and viral infections through mechanisms that affect bone metabolism and induce osteoporosis.

Hallmark Gene Sets analysis shows that these genes are mostly associated with apoposis, uv_response_dn, coagulation, allograft_rejection, pi3k_akt_mtor_ signaling, complement, inflammatory_response, xenobiotic_metabolism and uv_response_up (Supplementary file Figure 3C).

In addition, we further validated this by Metascape analysis. Functional enrichment results indicate that nicotine may affect multiple biological pathways by triggering osteoporosis, including cancer pathway, response to exogenous stimuli, hypoxic response, VEGFA/VEGFR2 signaling pathway, hormone-stimulated cellular response, AGE/RAGE pathway, MAPK cascade response modulation, lipid response, hormone level regulation, hematopoiesis, organ homeostasis, atherosclerosis, prostate cancer-related pathways, attention deficit hyperactivity disorder (ADHD) and autism spectrum disorder (ASD) pathways, glandular development, response to endotoxin, androgen receptor network in prostate cancer, mechanical stimulus response, and inflammatory response. Together, these pathways may explain the effects of nicotine on osteoporosis as well as other physiological and pathological processes (Supplementary file Figure 3D).

### Core target screening and analysis

To further screen and analyze the key targets, we imported the TSV format files obtained from the STRING database into Cytoscape 3.9.0 software and deeply analyzed the PPI network by the network topology analysis tool. Using the MCC, EPC, MNC, Radility, Clseness and Degree algorithms in the Cytoscape plug-in, the top 10 targets in the PPI network were selected respectively (Supplementary file Figures 4A–4F). Finally, 8 core targets were selected by taking the intersection of 6 algorithms (Supplementary file Figure 4G), which were SRC, BCL2, HIF1A, TNF, NFKB1, STAT3, ESR1 and CASP3.

### Molecular docking of nicotine and core target proteins

**Figure 2:**
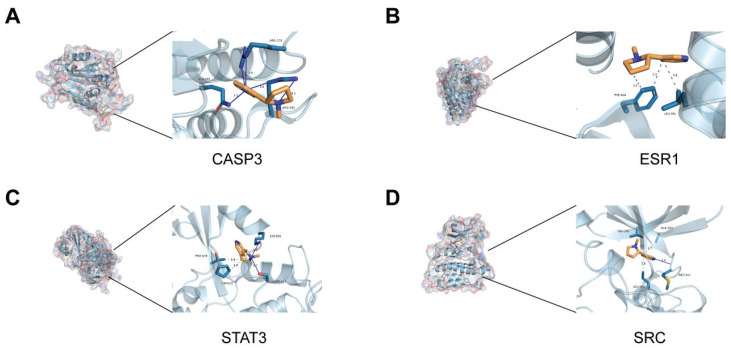
The results of molecular docking: A) Molecular docking conformation of nicotine with CASP3 protein; B) Docking complex of nicotine with ESR1 protein; C) Binding conformation of nicotine to STAT3 protein; D) Docking configuration of nicotine with SRC protein. Receptor proteins are in light blue, amino acid residues in dark cyan and red, nicotine molecules in orange and dark blue, and yellow lines indicating hydrogen bonds

To further validate the results of the network toxicology analysis, molecular docking experiments were performed. The docking results showed that the binding energies of the 8 target proteins with nicotine are approximately -5 kcal/mol, indicating strong binding affinity. On this basis, we chose the four proteins with the strongest ability to bind nicotine to osteoporosis to show in detail. The 3D binding models generated by PyMOL demonstrate that binding stability is enhanced through hydrophobic interactions, hydrogen bonds, perpendicular π-stacking, and salt bridges (Figures 2A–2D). The binding energies (kcal/mol) of the proteins are as follows: TNF= -5.6680, STAT3= -4.3471, SRC= -5.5375, NFKB1= -4.0290, ESR1= -5.5902, CASP3= -5.1505, and BCL2= -4.8661.

## DISCUSSION

In this study, 388 potential nicotine targets were identified using multiple prediction tools. Analysis of the osteoporosis dataset identified 1777 related genes, of which 116 overlapped with nicotine targets. GO and KEGG analyses indicated that nicotine may affect osteoporosis through pathways related to the effects of nicotine on osteoporosis, lipid metabolism and atherosclerosis, and apoptosis. Hallmark gene set analysis showed that these genes were mainly associated with apopos, uv_response_dn, etc. Metascape further confirmed that nicotine may affect osteoporosis by triggering multiple pathways such as cancer pathway, response to exogenous stimuli, hypoxic response, etc. PPI network analysis identified eight core targets: SRC, BCL2, HIF1A, TNF, NFKB1, STAT3, the four proteins with the strongest binding ability (CASP3, ESR1, STAT3, SRC) were analyzed by molecular docking.

These pathways do not act independently, and it was found that estrogen deficiency can inhibit osteoblast differentiation through the PI3K-Akt pathway^24^, while oxidative stress, activated through the NRF2 pathway, further promotes osteoclastogenesis through the NF-κB signaling pathway^25^. Meanwhile 8 core targets and nicotine have good binding activity, among which CASP3, ESR1, STAT3 and SRC have the strongest binding ability to it. CASP3 is a cysteine-aspartate protease involved in the process of apoptosis. As a key execution molecule of apoptosis, its overexpression can promote osteoblast apoptosis by activating the caspase cascade reaction^26^. In this study, CASP3 expression was up-regulated, suggesting that nicotine may exacerbate the progression of osteoporosis by inducing osteoblast apoptosis and inhibiting bone formation. ESR1 is an estrogen receptor, which is closely related to bone metabolism. Its downregulation is common in postmenopausal women with osteoporosis^27^. In this study, we found that nicotine significantly reduced the expression of ESR1, and the direct binding of nicotine to ESR1 was demonstrated by molecular docking analysis. This finding provides a molecular mechanism explanation for the correlation between smoking and osteoporosis in women, suggesting that nicotine exacerbates the risk of osteoporosis by interfering with the estrogen signaling pathway. STAT3, as a key molecule in the JAK-STAT signaling pathway^28^, and its phosphorylation level is closely related to osteoclast differentiation and bone resorption. In the present study, we found that nicotine activated this pathway by up-regulating the expression of STAT3, and further by up-regulating the expression of the osteoclast markers CTSK and TRAP^29^, corroborating that nicotine may exacerbate the pathological process of osteoporosis by promoting osteoclast activity. The Src protein tyrosine kinase (SRC) is an important non-receptor tyrosine kinase involved in multiple cell signaling pathways. The role of Src in osteoblasts is mainly focused on its high expression in osteoclasts. Osteoclasts are responsible for bone resorption and degradation, and Src activity is an essential part of osteoclast function, which is critical for the bone remodeling process^30^. In addition, Src-mediated signaling can also affect the activity of osteoblasts^31^, although the regulatory mechanisms differ somewhat and may produce different biological effects. The interaction of nicotine with Src may affect osteoblast function and thus interfere with the bone remodeling process.

Molecular docking analysis further validated the interactions between nicotine and multiple targets. These bindings were stabilized by forces such as hydrogen bonding and hydrophobic interactions between nicotine and the targets. In particular, nicotine binds to ESR1, resulting in a conformational change in its ligand-binding domain, which may regulate bone metabolism by affecting estrogen signaling pathways^32^. This finding provides new ideas for the development of therapeutic drugs targeting ESR1 and provides mechanistic support for understanding smoking-induced osteoporosis.

The studies listed above reveal that nicotine may affect osteoporosis through multiple biological pathways, provide an in-depth understanding of the mechanism of action of nicotine on osteoporosis, and identify CASP3, ESR1, STAT3, and SRC as potential core targets, which provide a theoretical basis for further research.

Although this study provides potential mechanisms for nicotine’s regulation of osteoporosis, the specific effects of nicotine on osteoporosis remain unclear. Previous studies have shown that nicotine has differential effects on osteoblasts depending on the dose: high doses of nicotine are toxic to cells, whereas low doses may promote cell proliferation^33^. Some studies suggest that low-dose nicotine could be used as a treatment for osteoporosis based on this premise^34^, while others oppose this view^35^. Additionally, there are studies indicating that nicotine does not have a significant impact on bone density or bone mass^36^. Therefore, the effects of nicotine on osteoporosis still require further validation.

### Limitations

The present study has several limitations. First, it primarily relies on bioinformatics analyses and *in vitro* cellular experiments, while lacking validation in animal models and clinical specimens, which may restrict the extrapolation of the findings to *in vivo* conditions. Second, the possible synergistic or antagonistic effects of other toxic components in tobacco smoke (such as tar and heavy metals) on bone metabolism were not fully addressed, potentially influencing the actual biological effects of nicotine. Third, although molecular docking offers useful predictions regarding the binding affinity between nicotine and target proteins, it remains a computational approach and may not accurately represent biological activity or functional outcomes in living systems. Finally, the current analysis was based on a single dataset without validation across multiple independent cohorts, which could compromise the robustness and generalizability of the results. Future studies should therefore integrate animal experiments, clinical samples, multi-dataset validation, and functional assays to strengthen the reliability of the conclusions.

### Future research

Future studies should be expanded to improve the physiological relevance and clinical translational value of the present study. On the one hand, the role of core targets and related signaling pathways *in vivo* can be verified by constructing nicotine-contaminated mouse models. On the other hand, the correlation between serum markers (e.g. CASP3, ESR1) and bone mineral density in the smoking population can be analyzed in conjunction with clinical cohort studies to assess the potential clinical effects of nicotine on osteoporosis. In addition, bone-protective drugs based on ESR1/ CASP3 dual targets should be explored to further evaluate their application prospects in osteoporosis prevention and treatment.

## CONCLUSIONS

Anchored in a network-toxicology framework and supported by multi-database target prediction, GEO transcriptomics, enrichment analyses, and molecular docking, this study delineates the molecular network linking nicotine to osteoporosis: among 388 nicotine-related targets and 1777 OP genes, 116 overlapped; PPI topology highlighted eight core nodes (SRC, BCL2, HIF1A, TNF, NFKB1, STAT3, ESR1, CASP3); enrichment underscored apoptosis and estrogen signaling as key pathways; and docking indicated favorable binding for several targets, with CASP3, ESR1, and SRC among the better binders. Collectively, the evidence supports a model in which nicotine exacerbates OP by promoting apoptosis, perturbing estrogen signaling, and engaging osteoclast-associated hubs. These nodes and pathways nominate testable biomarkers and intervention points for smoking-related OP and motivate *in vivo* and clinical validation.

## Supplementary Material



## Data Availability

The data supporting this research are available from the authors on reasonable request.
