# Supplementary material for: Unraveling the mechanisms of nicotine-induced osteoporosis via network toxicology, bioinformatics, and molecular docking
Source: Tob Induc Dis. 2026 Jan 20;24:10.18332/tid/215177. doi: 10.18332/tid/215177 (PMC12817853; doi:10.18332/tid/215177)
Supplement: Supplementary file 1 [file TID-24-06-s1.pdf]

Supplementary Table 1 Databases and tools with associated URLs

Databases and tools	URLs
PubChem	https://pubchem.ncbi.nlm.nih.gov/
SuperPred	https://prediction.charite.de/
Target Net	http://targetnet.scbdd.com/home/index/
PharmMapper	https://www.lilab-ecust.cn/pharmmapper/
ChEMBL	https://www.ebi.ac.uk/chembl/
SwissTargetPrediction	http://swisstargetprediction.ch/
Similarity Ensemble Approach	https://sea.bkslab.org/
ProTox-3.0	https://tox.charite.de
Gene Expression Omnibus	https://www.ncbi.nlm.nih.gov/geo/
GeneCards	https://www.genecards.org/
Comparative Toxicogenomics Database	https://ctdbase.org/
STRING	https://string-db.org/
Hiplot	https://hiplot.org
SangerBox	http://vip.sangerbox.com
Metascape	http://metascape.org
RCSB PDB	https://www.rcsb.org/

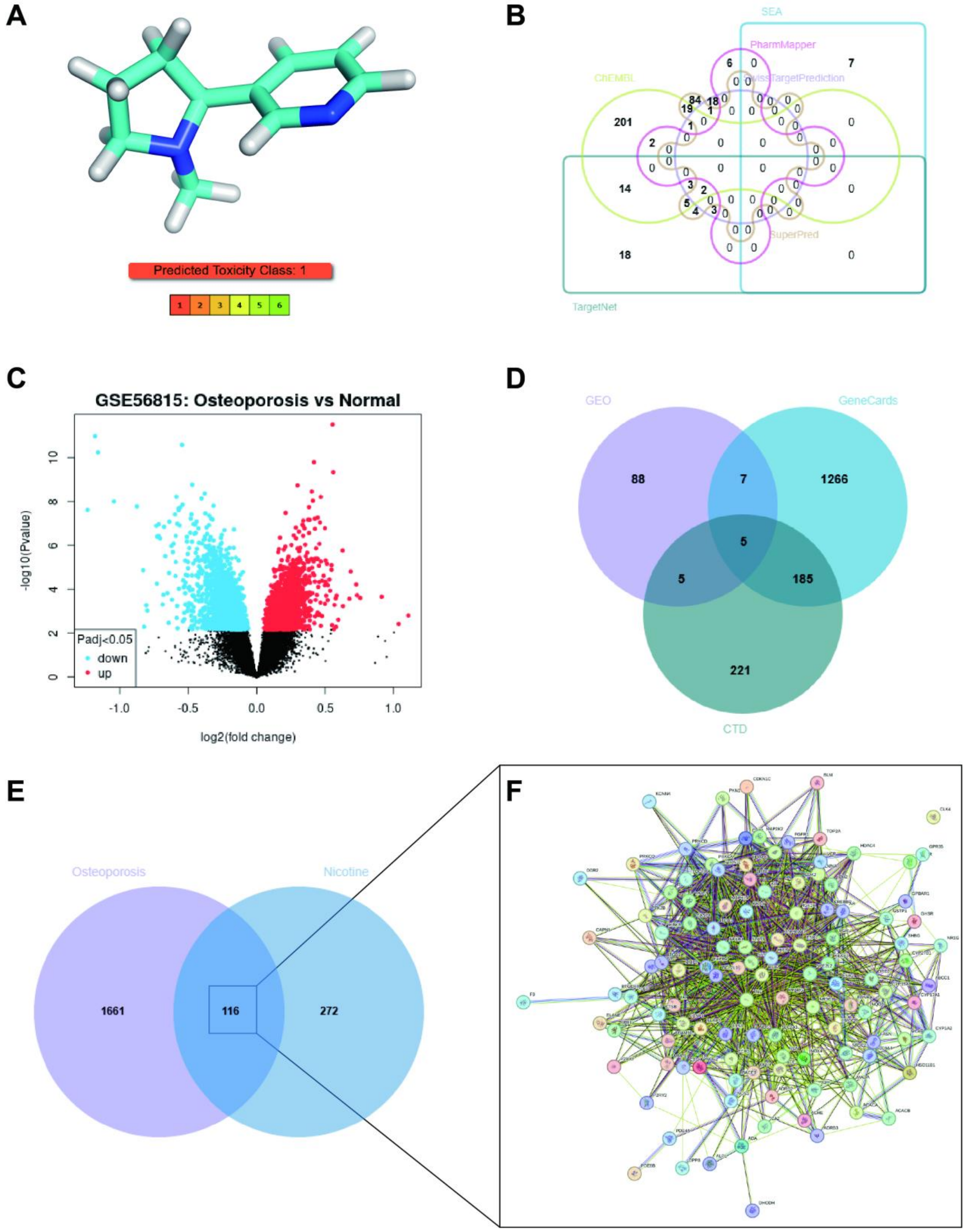

Supplementary Fig. 1. Prediction of nicotine toxicity and construction of PPI networks for intersecting targets with osteoporosis

A. 3D structure and toxicity prediction of nicotine. The three-dimensional structure of nicotine, with its predicted toxicity classification (Class 1) based on ProTox toxicity prediction tool.

B. Common target prediction. Venn diagram summarizing the protein targets predicted

by six independent databases; overlaps represent targets identified in multiple databases. A total of 388 potential targets were identified through the intersection of these sources.

C. Differential expression in osteoporosis. Volcano plot for the GSE56815 dataset comparing osteoporosis samples to normal controls. (x-axis: $\log_2$ (fold change); y-axis: $-\log_{10}$ (adjusted P-value); red: upregulated DEGs; blue: downregulated DEGs; n = 40; Padj < 0.05)

D. Identification of osteoporosis-related genes. Osteoporosis-related genes were screened from the GEO, CTD database, and GeneCards tool, followed by concatenation and removal of duplicates, resulting in 1,777 unique osteoporosis-related genes.

E. Common targets of nicotine and osteoporosis. Genes related to osteoporosis and nicotine exposure are illustrated using a Venn diagram, showing 116 common targets.

F. PPI network of nicotine and osteoporosis co-interactions. A PPI network of nicotine and osteoporosis co-interactions was constructed using the STRING database.

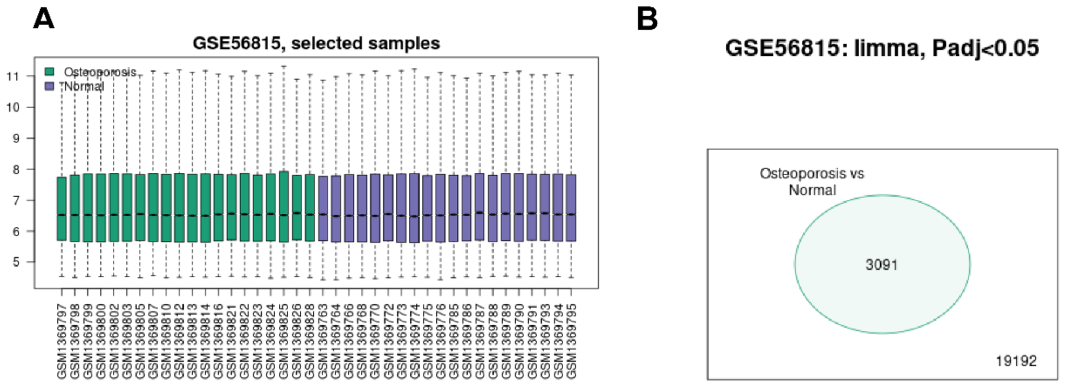

Supplementary Fig. 2. Standardization and Differential Expression Analysis of GSE56815

A. Box plot of normalized gene expression values. The x-axis represents sample IDs, and the y-axis represents normalized gene expression values, showing consistent median levels across all samples. The dataset GSE56815 consists of 40 samples, including 20 osteoporosis cases and 20 normal controls. The expression values were quantile-normalized to ensure comparability across samples.

B. Identification of differentially expressed genes. A total of 3,091 genes were found to

be differentially expressed between the osteoporosis and control groups. Statistical significance was evaluated using the limma package, which adjusts for multiple comparisons using the Benjamini-Hochberg method. Genes with an adjusted P-value < 0.05 were considered significantly differentially expressed.

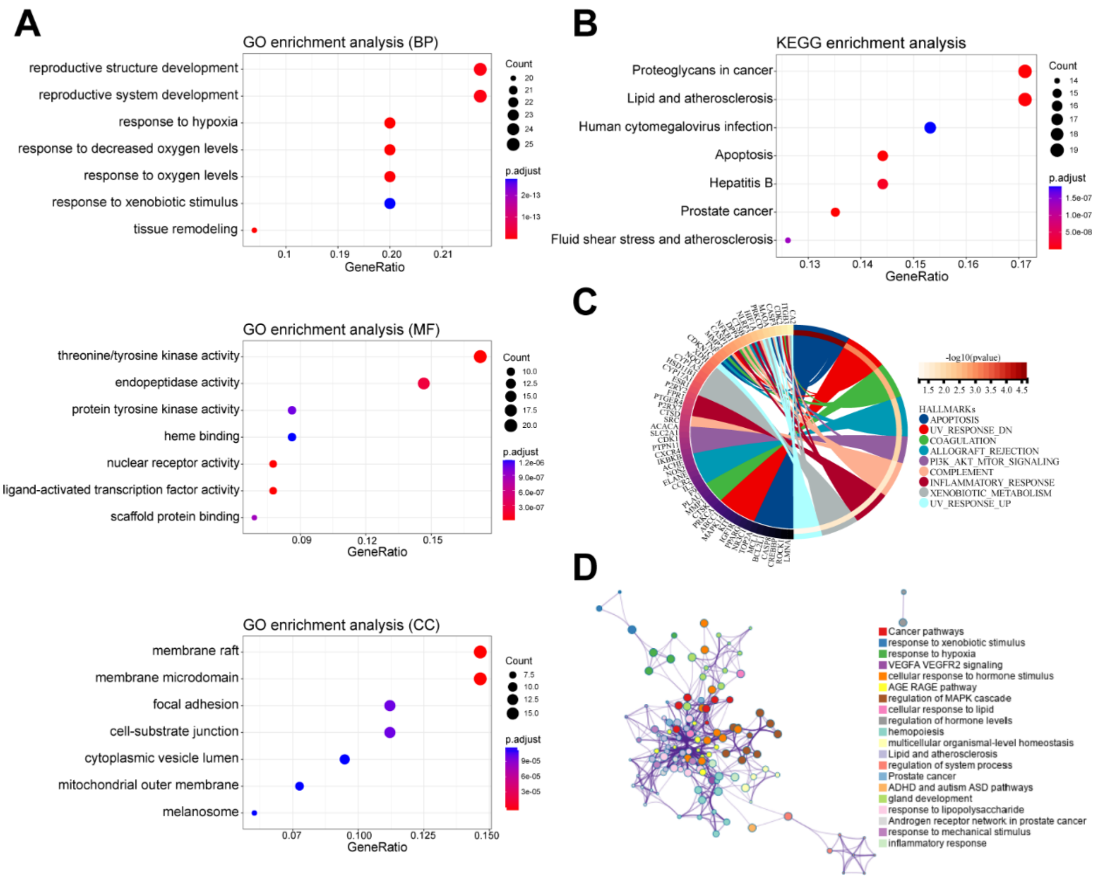

Supplementary Fig. 3. Functional enrichment and network visualization of DEGs

A. GO enrichment analysis. Bubble plots of GO (BP), GO (CC), GO (MF) functional analysis by GO enrichment analysis and the top 7 entries ranked by Count number. The x-axis shows the GeneRatio, bubble size represents gene count, and color encodes adjusted P-value.

B. KEGG pathway enrichment. Signaling pathways screened using KEGG pathway enrichment analysis. The top 7 entries ranked by gene count are shown as bar graphs, with bar color indicating adjusted P-value.

C. Chord diagram of Hallmark gene sets. The chord diagram displays selected pathways and their associated genes. Outer labels represent pathways and genes, while ribbons

illustrate gene-pathway associations. The inner color gradient represents -log10 (P value), with darker shades indicating higher enrichment significance and lighter shades indicating lower significance.

D. Term network. The term network displays representative terms selected from all clusters. Each term is represented as a circular node, with node size proportional to the number of input genes associated with that term, and node color indicating cluster identity (terms within the same cluster share the same color). Terms with pairwise similarity scores greater than 0.3 are connected by edges, with edge thickness reflecting the strength of similarity.

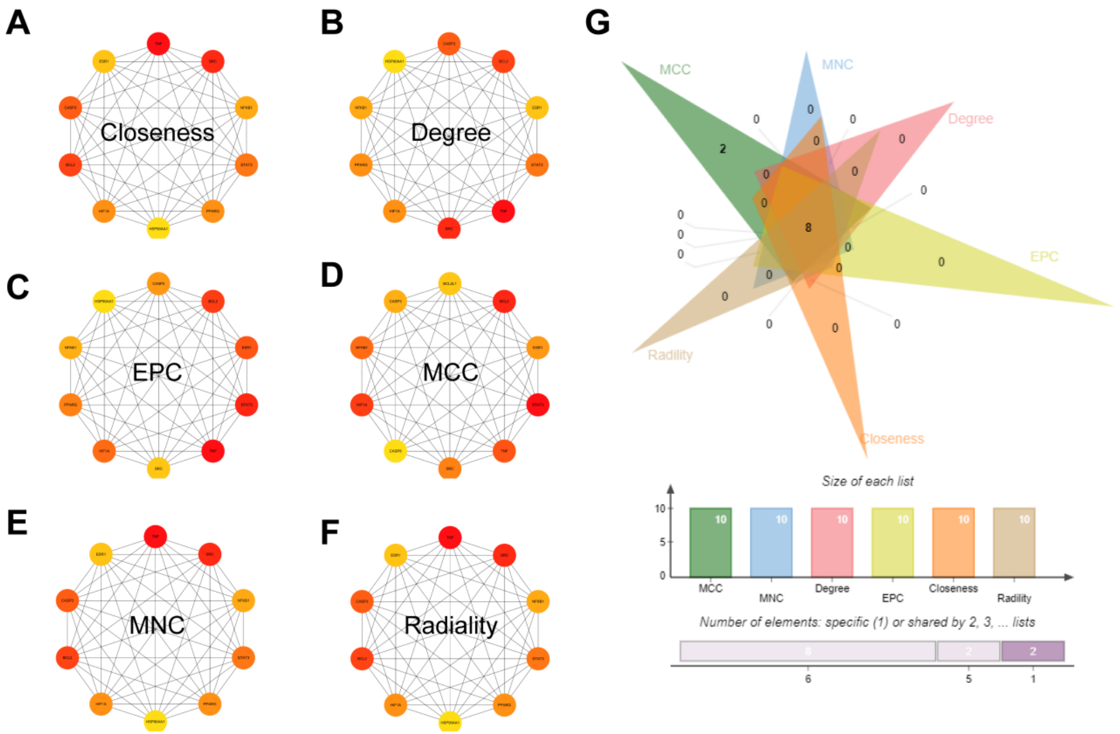

Supplementary Fig. 4. Analyze PPI networks to screen for core targets

A-F. Top 10 hub genes identified by different centrality metrics. Top 10 results from the analysis of centrality metrics, including MCC, EPC, MNC, Closeness Centrality, Radiality Centrality, and Degree.

G. Nodes in the network were evaluated using six different centrality metrics. Each attribute list contains 10 elements, and 8 elements appear in all lists simultaneously, leading to the identification of 8 core targets.

© 2026 Xu S. et al.
